# Lrrn1 is required for formation of the midbrain–hindbrain boundary and organiser through regulation of affinity differences between midbrain and hindbrain cells in chick

**DOI:** 10.1016/j.ydbio.2011.02.002

**Published:** 2011-04-15

**Authors:** Kyoko Tossell, Laura C. Andreae, Chloe Cudmore, Emily Lang, Uma Muthukrishnan, Andrew Lumsden, Jonathan D. Gilthorpe, Carol Irving

**Affiliations:** a Department of Cell and Developmental Biology, University College London, Gower Street, London, WC1E 6BT, UK; b MRC Centre for Developmental Neurobiology, Kings College London, London, SE1 1UL, UK; c Umeå Centre for Molecular Medicine, Umeå University, 901 87 Umeå, Sweden

**Keywords:** *Lrrn1*, Tartan, Capricious, Boundary, Midbrain–hindbrain boundary organiser

## Abstract

The midbrain–hindbrain boundary (MHB) acts as an organiser/signalling centre to pattern tectal and cerebellar compartments. Cells in adjacent compartments must be distinct from each other for boundary formation to occur at the interface. Here we have identified the leucine-rich repeat (LRR) neuronal 1 (Lrrn1) protein as a key regulator of this process in chick. The Lrrn family is orthologous to the *Drosophila* tartan/capricious (trn/caps) family. Differential expression of trn/caps promotes an affinity difference and boundary formation between adjacent compartments in a number of contexts; for example, in the wing, leg and eye imaginal discs. Here we show that *Lrrn1* is expressed in midbrain cells but not in anterior hindbrain cells. *Lrrn1* is down-regulated in the anterior hindbrain by the organiser signalling molecule FGF8, thereby creating a differential affinity between these two compartments. Lrrn1 is required for the formation of MHB — loss of function leads to a loss of the morphological constriction and loss of *Fgf8*. Cells overexpressing *Lrrn1* violate the boundary and result in a loss of cell restriction between midbrain and hindbrain compartments. Lrrn1 also regulates the glycosyltransferase *Lunatic Fringe*, a modulator of Notch signalling, maintaining its expression in midbrain cells which is instrumental in MHB boundary formation. Thus, Lrrn1 provides a link between cell affinity/compartment segregation, and cell signalling to specify boundary cell fate.

## Research highlights

*►Lrrn1* is expressed in midbrain cells but not in anterior hindbrain cells. *►Lrrn1* is down-regulated in anterior hindbrain by FGF8. *►Lrrn1* creates a differential affinity between midbrain and hindbrain compartments. ►This forms a sharp compartment boundary at the midbrain–hindbrain interface. ►Lrrn1 is required for formation of the mid–hindbrain boundary.

## Introduction

The MHB is an organising centre that is crucial for the formation of tectum and cerebellum from midbrain and hindbrain, respectively (Chi et al., 2003; Marin and Puelles, 1994; Martinez et al., 1995; Reifers et al., 1998). It arises at Hamburger Hamilton stage (HH)10 in the chick when morphological constrictions begin to appear along the length of the neural tube, sub-dividing it into smaller units upon which patterning signals can bestow specific regional identities (Lumsden and Krumlauf, 1996).

The formation of the MHB is a complex process, involving the integration of numerous signalling pathways. Early in development the position of the future boundary is demarcated by the expression borders of the homeobox transcription factors, *Otx2* and *Gbx2*, which abut at the interface between midbrain and hindbrain (Millet et al., 1996, 1999; Wassarman et al., 1997). Experimental manipulation of this expression interface results in a corresponding shift in the position of the MHB (Broccoli et al., 19990; Katahira et al., 2000; Millet et al., 1999). A number of genes have been identified with expression domains at the MHB, forming a large signalling network that generates the complexity required for production of a stable organiser signal. *Fgf8* is the best candidate for providing the MHB organiser signal, as recombinant FGF8 protein can mimic organiser tissue grafts when inserted into the neural tube (Crossley et al., 1996; Irving and Mason, 2000). *Fgf8* is first expressed in a broad domain at the MHB from HH8−. As the boundary forms, *Fgf8* becomes restricted to a tight domain on the posterior (hindbrain) side of the boundary (Shamim et al., 1999). Similarly, *Wnt1* is broadly expressed in midbrain but becomes progressively restricted to a stripe on the anterior (midbrain) side of the boundary (McMahon and Bradley, 1990). These and other genes become locked in a regulatory network that maintains and restricts the organiser at the boundary, through mutual positive and negative feedback loops (Canning et al., 2007; Wurst and Bally-Cuif, 2001; Ye et al., 2001).

Formation of the MHB also requires the action of the Notch signalling pathway (Tossell et al., submitted for publication). The Notch modifier, *Lunatic Fringe (LFng)*, is a glycosyltransferase that together with the distribution of Notch ligands, *Delta* and *Serrate*, is instrumental in determining where Notch signalling is active (Wu and Rao, 1999). At the MHB, we have recently shown that the border of *LFng* expression coincides with the *Otx2:Gbx2* border, where the MHB will form, and that the integrity of this border of *LFng* expression is instrumental for boundary formation. Furthermore, cells ectopically expressing activated Notch are excluded from the r1/2 domain (metencephalon), and instead are clustered at the MHB and r2/3 boundaries, where activated Notch promotes boundary cell fate (Tossell et al., submitted for publication).

Cellular affinity or adhesive differences between cells in adjacent compartments are necessary to help stabilise separate domains to allow a boundary to form at their interface. For example, either cells within compartments could have a high affinity for each other, or cells in adjacent compartments could repel each other, thereby preventing intermixing. In the hindbrain adhesive differences between rhombomere compartments drive cell sorting, and together with cell plasticity, lead to a stable interface which is necessary for boundary formation (Guthrie and Lumsden, 1991; Irving et al., 1996; Wizenmann and Lumsden, 1997). Ephrin/Eph receptor signalling is important for rhombomere compartment-specific cell sorting (Cooke et al., 2001; Mellitzer et al., 1999; Xu et al., 1999). At the interface of Ephrin/Eph expression domains, Notch signalling promotes the segregation of boundary cells from rhombomere compartments and inhibits neurogenesis (Cheng et al., 2004; Pan et al., 2004). These boundary cells are identified by their elongated morphology, fan-shaped arrangement, low rate of proliferation, lack of neurogenesis and the expression of a number of molecular markers (Guthrie and Lumsden, 1991; Trokovic et al., 2005). At the MHB, the midbrain and hindbrain form such compartments that do not mix, are lineage restricted and form a boundary at their interface that displays similar characteristics to hindbrain boundary cells (Jungbluth et al., 2001; Langenberg and Brand, 2005; Trokovic et al., 2005; Zervas et al., 2004). Notch signalling is implicated in the specification of boundary cell fate at the MHB (Tossell et al., submitted for publication). However, the mechanisms that prevent cell mixing across this boundary remain unknown.

In *Drosophila*, capricious (caps) and tartan (trn) are members of the LRR family. Both are transmembrane proteins characterised by a long extracellular domain containing 14 LRRs and a relatively short intracellular domain. Caps was originally identified due to its role in neuromuscular target recognition where it mediates the interaction between innervating axons and their targets, possibly through homophilic adhesion. *Caps* is expressed in both innervating axon and target muscle, and has been shown to bind homophilically in a cell aggregation assay (Shishido et al., 1998). Subsequently, caps has also been found to regulate axonal targeting in the visual system (Shinza-Kameda et al., 2006). In the leg imaginal disc, caps and trn may act to promote cell mobility within the epithelium, as overexpression leads to cell invasion of ectopic territories whilst down-regulation coincides with reduced mobility (Sakurai et al., 2007). Similarly in the eye, Mao et al. have recently proposed that caps and trn regulate adhesive properties that allow ommatidial organisation from the epithelial sheet (Mao et al., 2008).

*Caps* and *trn* are expressed in a compartment restricted fashion at the DV boundary of the wing imaginal disc where they prevent cell mixing between compartments (Milan et al., 2001). Both are regulated by the selector gene *Apterous (Ap)* and their expression only in dorsal cells mediates an affinity difference between dorsal and ventral cells, preventing cell mixing between adjacent compartments (Milan and Cohen, 2003; Milan et al., 2001, 2005). Forced expression of *caps* or *trn* in ventral cells is sufficient to cause cells to sort to the dorsal compartment (Milan et al., 2001). Furthermore, in an *Ap* mutant background where the DV boundary is lost, expression of *caps* or *trn* is sufficient to rescue the affinity boundary (Milan et al., 2001). It is unclear whether trn and caps function through homophilic adhesion here as in other systems, or whether they act as ligands in an as yet unknown pathway. However, no evidence of a receptor has been found to date (Andreae et al., 2007; Milan et al., 2005; Shishido et al., 1998).

The closest vertebrate orthologues of *trn* and *caps* are members of the LRR family: *Lrrn1*, *Lrrn2* and *Lrrn3* (Taguchi et al., 1996; Taniguchi et al., 1996). *Lrrn1* has recently been identified in chick, as a 3′ EST (2B10) isolated from a subtracted hindbrain cDNA library, with expression in the midbrain and posterior hindbrain from the r2/3 boundary, but specifically absent from the MHB at HH10 (Christiansen et al., 2001). More recently, a full length chick *Lrrn1* cDNA has been identified (Andreae et al., 2007; Garcia-Calero et al., 2006). Lrrn1 has 12 LRRs flanked by characteristic cysteine-rich repeats, an immunoglobulin-like (Ig-like) domain and a fibronectin type III domain. The intracellular domain contains two endocytic sorting motifs and a PDZ ligand binding motif.

Intriguingly, *Lrrn1* has a dynamic expression pattern in the CNS, with specific down-regulation at boundaries, the timing of which correlates with the activation of signalling molecules there (Andreae et al., 2007). *Lrrn1* expression demarcates the position of the anterior boundary of the zona limitans intrathalamica (ZLI), becoming down-regulated just before the onset of the signalling molecule *Shh* within the ZLI. In the hindbrain, *Lrrn1* is down-regulated at rhombomere boundaries as they become morphologically distinct. Similarly, at the MHB the down-regulation of *Lrrn1* in r1/2 correlates with the onset of MHB organiser gene expression and boundary formation (Andreae et al., 2007).

Due to the striking expression pattern of *Lrrn1* with respect to boundary regions in the CNS and the role of caps/trn in boundary formation, we hypothesised that Lrrn1 may be involved in establishing the compartment boundary between midbrain and hindbrain at the MHB. Here we show that the posterior border of *Lrrn1* expression correlates with the posterior midbrain border at the MHB, where it is co-expressed with *LFng* and *Otx2*. Reducing Lrrn1 function using Morpholinos or the overexpression of truncated proteins disrupts this border and results in loss of the organiser, as assessed by *Fgf8* expression. Misexpression of *Lrrn1* across the boundary coupled with cell lineage labelling reveals that the border of *Lrrn1* expression is important for making an affinity boundary between midbrain and hindbrain cells. Furthermore, disrupting the border by ectopically expressing *Lrrn1* in anterior hindbrain causes midbrain and hindbrain cells to mix and disrupts organiser activity. *Lrrn1* regulates *LFng* expression and is itself regulated by FGF8, providing a link between compartment segregation, boundary formation and organiser signalling. Although the mechanism of action of this gene family is currently unknown, here we show that Lrrn1 does not act as a homophilic adhesion molecule to prevent cell mixing.

## Materials and methods

### Chick embryos

Fertile chick eggs (Brown Bovan Gold; Henry Stewart & Co.) were incubated at 38 °C and staged according to Hamburger and Hamilton (Hamburger and Hamilton, 1992).

### In ovo electroporation of DNA constructs

Hamburger and Hamilton stage (HH) 8–9 chick embryos were electroporated using fine platinum electrodes and an ElectroSquare Porator™ ECM 830 (BTX) on the following settings; 25 V, 4 pulses, 50 ms duration, interval 950 ms. DNA was injected into the neural tube; electrodes were placed on the vitelline membrane either side of the neural tube.

A full length chicken *Lrrn1* cDNA, under the control of the chicken ß-actin promoter, was linked with green fluorescent protein (GFP) by an internal ribosomal entry site (IRES-GFP): Lrrn1-IRESGFP (1 μg/μl). Construct Lrrn1T-GFP carries a truncation immediately after the predicted transmembrane domain at amino acid 655, which is fused to the N-terminus of GFP. Consequently it lacks the intracellular portion of the protein including the putative C-terminal PDZ interaction and endocytosis motifs (1 μg/μl). Mouse *LFng*-IRESGFP (1 μg/μl) was a kind gifft from O. Cinquin. An anti-sense Morpholino oligonucleotide was targeted to a region surrounding the ATG initiation codon of the *Lrrn1* mRNA in order to block translation of the LRRN1 protein. The *Lrrn1* Morpholino was tagged with fluorescein isothiocyanate (FITC) to enable detection within the embryo and facilitate uptake of the uncharged molecule by electroporation.

### In situ hybridisation and immunohistochemistry

In situ hybridisation used digoxygenin (DIG) and FITC labelled probes as previously described (Irving and Mason, 2000). Both probes were added simultaneously. Alkaline phosphatase (AP)-conjugated anti-DIG and anti-FITC antibodies (Roche) were added sequentially. DIG probes were detected using NBT:BCIP (Roche); FITC probes were detected using FAST TR/Naphtol AS-MX solution (Sigma). Embryos were fixed in 4% paraformaldehyde for 20 min before immunohistochemistry as previously described (Irving et al., 2002). Anti-EGFP antibody (rabbit polyclonal: Clontech) was used at 1:1000. Secondary anti-rabbit HRP was used at 1:200. Embryos were flatmounted or sectioned as previously described (Irving and Mason, 2000).

### Iontophoresis

In ovo electroporation was carried out with the appropriate GFP constructs at HH8+,9, then DiI (Molecular Probes; D-282) was immediately applied by iontophoresis (Nittenberg et al., 1997).

### Bead implantation

FGF8b was introduced on heparin-coated acrylic beads. Control beads were soaked in PBS. Beads were implanted as previously described (Irving and Mason, 2000).

### Cell sorting assay

Assays were performed using human embryonic kidney 293T (HEK293T) cells as described previously (Karaulanov et al., 2006). Cells were transiently transfected at 50–70% confluence in a 6-well plate with 200 ng of pCAß-eGFPm5 (Yaneza et al., 2002) and 1.8 μg per well of the specific expression vector being assayed (see below), using Fugene HD (Roche). A ratio of 6 μl Fugene HD:2 μg DNA resulted in a transfection efficiency of 80–100% as judged by eGFP fluorescence. After 24 h, cells were trypsinised, mixed with an equal number of control cells transfected with 200 ng of pCAß-mRFP1 and 1.8 μg per well of pUC19 and allowed to aggregate in complete medium in bovine serum albumin (BSA)-coated 48-well plates (coated with 1% (w/v) BSA in PBS, overnight at 4 °C) on an orbital shaker (120 R.P.M.). After 48 h, cell aggregates were examined on an inverted microscope (Zeiss Axiovert with YFP and CY3 filter sets) and photographed with a Zeiss Axiocam HS camera. Images were pseudocoloured and superimposed in Image J. The expression vectors used were: pCS2+−xFLRT3 and pCS2+−xFLRT3ΔLRR (positive and negative controls, respectively. Karaulanov et al., 2006), Flag-mLrrn1, N-terminally Flag-tagged mouse *Lrrn1* in pCDNA3.1(+) (Haines et al., 2005). CAß-mRFP1 was made by cloning a *BamH*I-*EcoR*I fragment of pRSETB-mRFP1 (Campbell et al., 2002) in to pCAß.

## Results

### Spatio-temporal expression of *Lrrn1* at the MHB

**Fig. 1:**
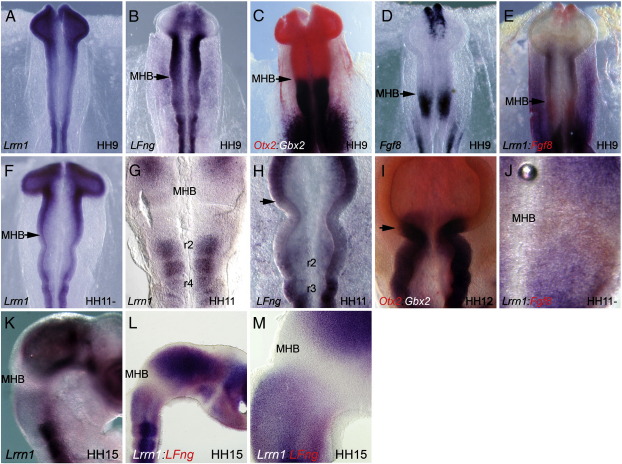
Expression of Lrrnl during chick MHB development. Wholemount in-situ hybridization of Lrrnl, LFng, Otx2 (red), Gbx2, Fgf8 (red). Neural tubes in (G,J) are opened in a flatmount preparation. (A) At HH9 Lrrnl begins to be down-regulated in the MHB region with high levels of expression remaining in the anterior and posterior neural tube. (B) LFng is also down-regulated in the MHB region at HH9. (C) The MHB is defined by the expression interface of Otx2 and Gbx2. (D) Fgf8 is expressed in the anterior-most domain of Gbx2 expression at the MHB. (E) Double in-situ hybridisation of Lrrnl (blue) and Fgf8 (red) show that Lrrnl is down-regulated in the Fgf8 domain. (F) At HH11− there is clear down-regulation of Lrrnl in MHB. (G)Absence of Lrrnl in MHB and also in rhombomere boundaries in hindbrain at HH11. (H) LFng expression mirrors that of Lrrnl at HH11 and is down-regulated at the MHB. (I) The expression border of Otx2 and Gbx2 is anterior to the morphological constriction at HH12 and coincides with the posterior border of LFng expression (black arrows). (J) Double in-situ of Fgf8 (red) and Lrnn1 (blue) shows complementary expression at the MHB at HH11−. (K) Transverse view of Lrrnl at HH15 shows clear down-regulation in MHB and a decreasing gradient in posterior midbrain towards the MHB. (L) LFng (red) and Lrnn1 (blue) are expressed coincidently in midbrain and hindbrain and are both absent at the MHB. (M) High magnification view of MHB in (L) showing coincident expression of LFng (red) and Lrrnl (blue). MHB: mid-hindbrain boundary, r2: rhombomere 2; r3: rhombomere 3: r4: rhombomere 4.

In order to investigate the expression of *Lrrn1* in the chick MHB in more detail, we performed a series of whole mount in-situ hybridisations on embryos between the 4 and 25 somite stages (HH8−15), over the time period that the midbrain and hindbrain compartments form. *Lrrn1* is expressed in the neural plate from HH5 (Andreae et al., 2007). We detected strong expression of *Lrrn1* throughout the neural plate at HH8 when the neural folds have formed. From stage HH9 *Lrrn1* was down-regulated in the MHB region (defined as the region surrounding the constriction where *Fgf8* is broadly expressed prior to boundary formation), with high levels remaining both anteriorly and posteriorly in the neural tube (Fig. 1A). Down-regulation was reminiscent of that of *LFng,* which becomes down-regulated in the MHB from HH9 (Fig. 1B). We have previously shown that this border of *LFng* expression coincides with the *Otx2*:*Gbx2* border (Tossell et al., submitted for publication), which defines where the MHB will form (Fig. 1C; Millet et al., 1996; Wassarman et al., 1997). The MHB organiser marker, *Fgf8,* is expressed in anterior hindbrain, with an anterior border of expression that coincides with *Gbx2* and abuts the *Otx2*-positive midbrain domain (Hidalgo-Sanchez et al., 1999) (Fig. 1D). Double in-situ hybridisation of *Fgf8* and *Lrrn1* revealed that *Lrrn1* was being down-regulated within this *Fgf8-*positive domain (Fig. 1E).

Down-regulation became increasingly marked as development proceeded and at HH11− there was an absence of *Lrrn1* in the posterior midbrain vesicle and anterior hindbrain (Fig. 1F). Flatmount analysis of the dissected neural tube revealed that *Lrrn1* was also absent from rhombomere boundaries at this stage as has previously been reported at later stages (Fig. 1G) (Andreae et al., 2007). The posterior border of *Lrrn1* expression at HH11 coincided with the posterior border of *LFng* and *Otx2:Gbx2* (Figs. 1H,I; Andreae et al., 2007). Double in-situ hybridisation analysis of *Fgf8* and *Lrrn1* revealed that the two genes were expressed in complementary, non-overlapping, domains at the MHB and in posterior r1 (Fig. 1J). Later in development *Lrrn1* expression could be seen in the midbrain, with a decreasing gradient of expression towards the MHB where it remained down-regulated. In the hindbrain *Lrrn1* was weakly expressed in r1, then strongly expressed in posterior rhombomeres (Fig.1K). The down-regulation at the MHB coincided with that of *LFng* (Figs. 1L,M). Therefore, *Lrrn1* also appears to define the extent of the midbrain compartment.

### FGF8 represses expression of *Lrrn1* at the MHB

**Fig. 2:**
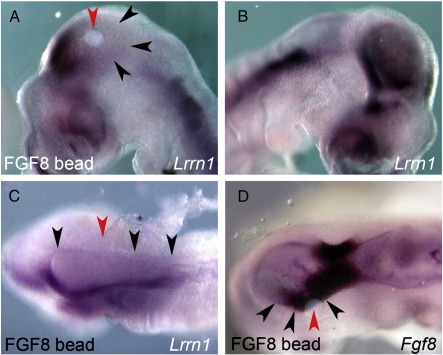
FGF8 represses Lrrnl expression at the MHB. (A) Lrrnl expression is down-regulated (black arrowheads) around an FGF8 bead inserted into midbrain. (B) Control contra-lateral side of the same embryo showing normal Lrnn1 expression throughout the midbrain. (C) Lrnn1 expression is down-regulated along the length of the hindbrain by an FGF8 bead inserted into hindbrain (black arrowheads). (D) Positive control: Fgf8 expression is up-regulated around an FGF8 bead inserted into midbrain (black arrowheads). Red arrowheads mark the position of the bead.

*Fgf8* is first expressed broadly in the MHB region at the 4 somite stage (HH8), shortly before *Lrrn1* becomes down-regulated there. At the 11 somite stage (HH10+) *Fgf8* is restricted to a tight domain at the MHB. *Lrrn1* was absent from this domain but continued to be expressed in the midbrain and hindbrain on either side. As *Lrrn1* appeared to be down-regulated at the MHB concurrent with the appearance of *Fgf8*, we investigated whether FGF8 was responsible for this dynamic expression. We introduced a local source of ectopic FGF8 protein unilaterally into the midbrain on heparin-coated acrylic beads at 7–8 somites, and examined the effect on *Lrrn1* expression. *Lrrn1* was dramatically down-regulated in the midbrain tissue surrounding the FGF8 bead (Fig. 2A and Table 1) as compared to the control contra-lateral side or to control beads soaked in PBS (Fig. 2B and Table 1). FGF8 beads implanted in the hindbrain were able to repress *Lrrn1* throughout the entire hindbrain (Fig. 2C). FGF8 induces *Fgf8* expression in the midbrain (Crossley et al., 1996; Martinez et al., 1999; Shamim et al., 1999). In agreement with this we observed strong induction of *Fgf8* around FGF8 beads (positive control) (Fig. 2D and Table 1). No induction of *Fgf8* was observed following implantation of PBS beads (Table 1). These results demonstrate that FGF8 downregulates *Lrrn1* expression and suggest that FGF8 may act normally to define the limits of *Lrrn1* expression in the posterior midbrain and anterior hindbrain, through repression in r1.

### Lrrn1 is required for boundary and organiser formation at the MHB

**Fig. 3:**
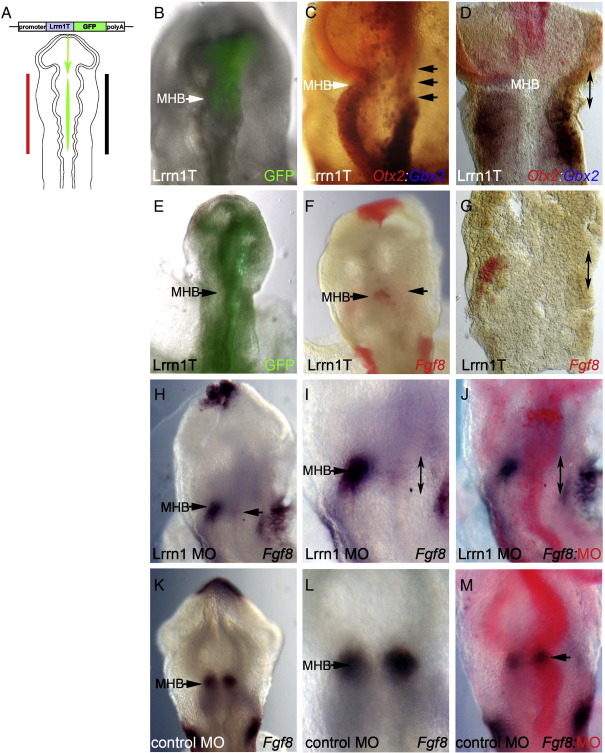
Lrrn1 is required for MHB boundary formation and organiser gene expression. (A) Schematic diagram of electroporation strategy into HH8+ or 9 chick embryos, and analysis 24 h later. (B–G) Electroporation of Lrrn1T-GFP. (B) Overlay of Lrrn1TGFP fluorescence reveals the position of electroporated cells. (C,D) In-situ hybridisation of *Otx2* (red) and *Gbx2* (blue) mark the midbrain and hindbrain compartments respectively. The neural tube appears flat on the electroporated right hand side and a diminished morphological constriction can be seen at the MHB (black arrows). (D) Flat mount preparation of the neural tube reveals that the sharp boundary between *Otx2* and *Gbx2* expressing cells is lost on the electroporated right hand side — compare to the control left hand side (black double arrow). (E) Overlay of Lrrn1TGFP fluorescence. (F,G) In-situ hybridisation of *Fgf8* (red). *Fgf8* is absent at the MHB on the electroporated right hand side of the neural tube. (G) Flat mount preparation of neural tube confirms the absence of *Fgf8* at the MHB on the electroporated side compared to the control contra-lateral side (black arrow). (H-J) Lrrn1 Morpholino (MO). (H) *Fgf8* expression (blue) is absent from the electroporated side of the neural tube at the MHB (black arrow) but remains expressed in other locations e.g. nasal placode. (I) High magnification confirms loss of *Fgf8* only on the electroporated side compared to the control contra-lateral side (black double arrow). (J) Anti-FITC antibody (red) reveals the position of the Morpholino within the neural tube. (K-M) control Morpholino. (K) *Fgf8* is expressed normally at the MHB (black arrow) and other locations. (L) *Fgf8* is expressed equally on both sides of neural tube in control embryos. (M) Anti-FITC antibody (red) reveals the position of the control Morpholino within the neural tube. MHB; midbrain–hindbrain boundary.

The posterior border of *LFng* expression in the midbrain is important for boundary formation at the MHB, as perturbation of the *LFng* expression border through ectopic expression in anterior hindbrain leads to a shift in position of the markers *Otx2*:*Gbx2*, and subsequently in the boundary itself (Tossell et al., submitted for publication). In order to investigate the involvement of Lrrn1 in compartition and boundary formation at the MHB we sought to block Lrrn1 function during development of the MHB boundary region, using a number of strategies. Firstly, we designed a construct containing a truncation of the intracellular domain, including the putative C-terminal PDZ interaction and endocytosis motifs, that could potentially work as dominant negative protein (Lrrn1T-GFP). Secondly, we used an anti-sense Morpholino oligonucleotide targeted to *Lrrn1* mRNA in order to block translation of the Lrrn1 protein following electoporation in vivo (Fig. 3A).

Electroporation of the truncated construct Lrrn1T-GFP into the neural tube of chick embryos at 5–7 somites (HH8+,9 — before the MHB has formed) resulted in an apparent loss of the morphological constriction at the MHB 24 h later. The neural tube was much straighter on the electroporated side compared to the contra-lateral control side (Fig. 3B,C,I). Using *Otx2* and *Gbx2* to mark the interface of midbrain and hindbrain compartments, we saw a caudal shift in *Gbx2* expression and a fuzzy interface between the two expression domains (Fig. 3B,C; n = 9/10) as compared to the control contra-lateral side or control embryos electroporated with GFP alone (Fig. 3B,C and data not shown; GFP control n = 0/7). We repeated these experiments using *Fgf8* as a molecular marker of the organiser itself, as recombinant FGF8 protein can mimic organiser grafts in vivo (Crossley et al., 1996; Irving and Mason, 2000). *Fgf8* expression at the MHB was either severely down-regulated, or in some cases, completely absent on the electroporated side (Fig. 3E–G; n = 8/10). In stark contrast we saw no effect on the control contra-lateral side of electroporated embryos or in controls electroporated with a GFP construct alone (Fig. 3E–G and data not shown; GFP control n = 0/10).

In order to confirm that the organiser was lost/disrupted through blocking Lrrn1 function we used an alternative strategy. We electroporated anti-Lrrn1 Morpholino into embryos at HH8+,9. *Fgf8* expression was found to be absent consistently, 24 h later on the side of the embryo where the Morpholino was detected using the FITC tag (Fig. 3H–J; n = 8/19). Furthermore, the morphology of the neural tube at the MHB was again perturbed, being much straighter than the control side (Fig. 3H–J). A standard fluorescent control Morpholino had no effect on *Fgf8* expression or MHB morphology (Fig. 3K–M; n = 10/10).

These data suggest that Lrrn1 is required for MHB boundary formation. Interfering with Lrrn1 function using a construct lacking the intracellular domain or by Morpholino-mediated knockdown elicited loss of a morphological constriction and downregulation of the organiser gene, *Fgf8*. This strongly suggests that the mutant construct acts as a dominant negative, and that the actions of Lrrn1 at the MHB are dependent on the presence of an intact intracellular domain.

### Ectopic expression of *Lrrn1* across the MHB causes loss of restriction of MHB gene expression borders and a shift in the morphological boundary

Previous studies in *Drosophila* have shown that caps and trn restrict cell movement across boundaries. Their ectopic expression across the border at both the DV boundary in the wing disc and tarsus 5-pretarsus boundary in the leg disc causes cell invasion of adjacent territories (Milan et al., 2001, 2005; Sakurai et al., 2007). The mechanism(s) involved remains unknown, but may be a direct result of a change in cell adhesive properties conferred by caps and trn.

**Fig. 4:**
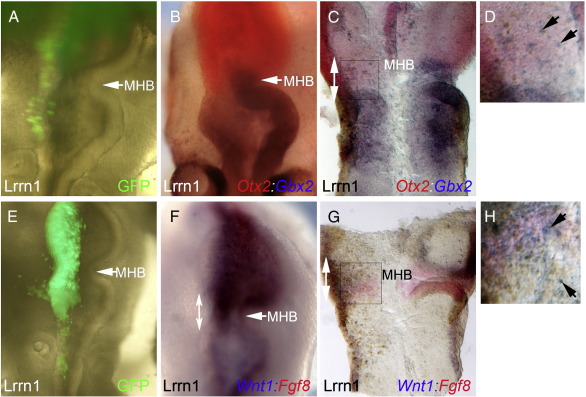
Ectopic expression of Lrrn1 across the MHB causes mixing at gene expression borders and a shift in the MHB. (A–H) Electroporation of Lrrnl-IRESGFP across the MHB interface (A) Overlay of Lrrnl-IRESGFP fluorescence reveals the position of electroporated cells. (B–D) In-situ hybridisation of Otx2 (red) and Gbx2 (blue). (B) The MHB morphological constriction and the expression border of Otx2 and Gbx2 are shifted caudally on the electroporated left hand side as compared to the control side (C) Flatmount preparation confirms the shift in expression of Otx2 and Gbx2 (white double arrow). (D) High magnification of box in C reveals mosaic expression of Obc2 and Gbx2 within anterior hindbrain (arrows). (F–H) In-situ hybridisation of Fgf8 (red) and Wntl (blue). Wntl marks anterior MHB in midbrain (Otx2-positive) cells FgfB marks posterior MHB in hindbrain (Gbx2-positive) cells (E) Overlay of Lrrnl-IRESGFP fluorescence. (F) Wntl expression extends caudally into anterior hindbrain on the electroporated left hand side as compared to control contra-lateral side (white double arrow) (G) The border of expression between Fgf8 and Wntl is fuzzy on the electroporated side (white double arrow). (H) High magnification of box in G reveals that Wntl-positive cells mix with Fgf8-positive cells and extend ectopically into the hindbrain (arrows). MHB: midbrain-hindbrain boundary.

To investigate the hypothesis that Lrrn1 confers a differential affinity property to cells in neighbouring compartments at the MHB, we ectopically expressed *Lrrn1* across the midbrain and hindbrain compartments, at the time of its normal down-regulation in anterior hindbrain. We electroporated an expression vector encoding full length *Lrrn1* cDNA into the neural tube at HH8+, 9 and analysed embryos 24 h later, when the MHB had formed. Cells ectopically expressing *Lrrn1* were visualised by co-expression of GFP from a bicistronic message linked by an internal ribosome entry sequence (Lrrn1-IRESGFP, Fig. 4A).

We used the molecular markers of midbrain and hindbrain compartments, *Otx2* and *Gbx2*, to analyse the effect of *Lrrn1* misexpression on compartition. When *Lrrn1* expression extended into the anterior hindbrain, crossing the compartment boundary, the expression border of *Otx2* and *Gbx2* was shifted caudally when compared to the control side, which was normal (Fig. 4A,B). *Otx2* positive cells were seen in rhombomere one. The morphological constriction of the MHB also shifted caudally on the electroporated side as compared to the control contra-lateral side, and became less well defined (Fig. 4A,B). Opening the neural tube in a flatmount preparation confirmed the shift in expression of *Otx2* and *Gbx2*, and revealed that cells were mosaically expressing *Otx2* and *Gbx2* within the anterior hindbrain (Fig. 4C,D; n = 5/5). We repeated these experiments using *Wnt1* and *Fgf8* as molecular markers of the organiser itself. *Wnt1* is normally restricted to a tight band of expression in anterior MHB in midbrain, *Otx2* positive cells, whereas *Fgf8* is restricted to a tight band of expression in posterior MHB in hindbrain, *Gbx2* positive cells. In embryos ectopically expressing *Lrrn1* across the boundary, *Wnt1* expression was seen extending caudally into anterior hindbrain. The border of expression between *Fgf8* and *Wnt1* was fuzzy on the electroporated side of the embryo, and *Wnt1* cells were observed mixing with *Fgf8*-positive cells (Fig. 4E–H; n = 4/5).

These results show that ectopic *Lrrn1* expression results in a loss of restriction of midbrain (*Otx2*) and hindbrain (*Gbx2*) markers and subsequently of MHB organiser genes *Wnt1* and *Fgf8*. Moreover, they suggest that coordinated down-regulation of *Lrrn1* from the MHB is required for boundary formation and appropriate specification of the organiser.

### Cell mixing at the midbrain–hindbrain compartment boundary

**Fig. 5:**
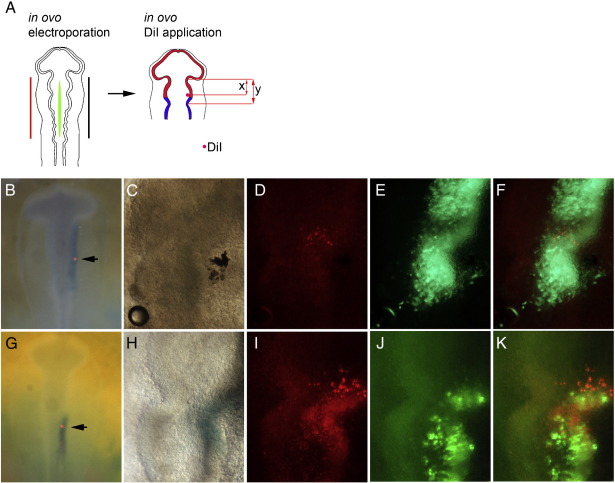
Misexpression of Lrnn1 across posterior midbrain and anterior hindbrain allows cells to move across the MHB. (A) Schematic representation of electroporation followed immediately by iontophoresis of Dil label (x) at 6/8th of (y), the distance along the neural tube from diencephalon to hindbrain constriction (just anterior to Otx2:Gbx2 interface) at HH9. (B,G) Position of Dil label at Ohr. (B–F) GFP control; dorsal view. (C) Neural tube 24 h later. (D) Cells labelled with Dil after 24 h are clustered in posterior midbrain and do not cross the MHB boundary. (E) Control GFP expressing cells are continuous throughout posterior midbrain, MHB and anterior hindbrain, (F) Merged view of Dil labelled cells and GFP expressing cells. (G–K) Lrrnl-IRESGFP; dorsal view. (H) Neural tube 24 h later. (I) Cells labelled with Dil are observed extensively in both midbrain and hindbrain compartments 24 h after labelling. (J) Lrnn1-IRESGFP expressing cells are seen in both midbrain and crossing the MHB into hind-brain. (K) Merged view shows Dil labelled cells within the Lrrnl-electroporated domain in both midbrain and hindbrain. MHB: midbrain–hindbrain boundary; black arrows indicate position of Dil label.

Blocking Lrrn1 function at the MHB resulted in loss of a clear border of *Otx2:Gbx2* expression, loss of a morphological boundary and loss of *Fgf8* “organiser” expression. Ectopic expression of *Lrrn1* resulted in a caudal shift in the morphological boundary which correlated with a shift in the *Otx2:Gbx2* interface. This suggests that differential expression of *Lrrn1* at the MHB is required for proper boundary positioning and formation. We hypothesised that Lrrn1 may confer a cell surface affinity property to cells that is required to prevent cell mixing between midbrain and hindbrain compartments. Down-regulation of *Lrrn1* in hindbrain cells may be required to separate midbrain and hindbrain cells. Alternatively, the role of *Lrrn1* may be to specify midbrain fate; ectopic *Lrrn1*-expressing cells may induce midbrain-specific markers *Otx2* and *Wnt1*, and the cell mixing observed may be due to the mosaic nature of electroporation in the neural tube. To test between these two possibilities, we used DiI labelling to trace the movement of cells immediately following electroporation with Lrrn1-IRESGFP (Fig. 5A). A small number of midbrain cells just anterior to the *Otx2*:*Gbx2* interface, which marks the molecular boundary, were labelled with DiI by iontophoresis and analysed 24 h later. The DiI was positioned at (x), 6/8th of (y), the distance along the neural tube from diencephalon to hindbrain constriction (Fig. 5A,B,G). In control embryos electroporated with a GFP expression vector before DiI labelling, the progeny of labelled cells could be seen in a tight cluster in the anterior MHB and clearly within the electroporated domain (Fig. 5C–F; n = 18/18). In sharp contrast, however, in embryos electroporated with Lrrn1-IRESGFP, DiI labelled midbrain cells were seen extending throughout the MHB region and into r1. The extent of the DiI label was as extensive as the ectopic Lrrn1-IRESGFP domain, indicating that cells were free to move within the *Lrrn1*-positive domain but remained within it (Fig. 5H–K; n = 14/17). This data suggests that it is unlikely that Lrrn1 confers a cell-fate change on hindbrain cells to that of a midbrain fate. Rather, it is more likely that the boundary of expression of *Lrrn1* is important for determining how cells segregate at the MHB, and that the role of Lrrn1 at the MHB is to maintain the midbrain compartment and prevent cell mixing between domains.

### Lrrn1 regulates Notch signalling by positioning the *LFng* boundary

**Fig. 6:**
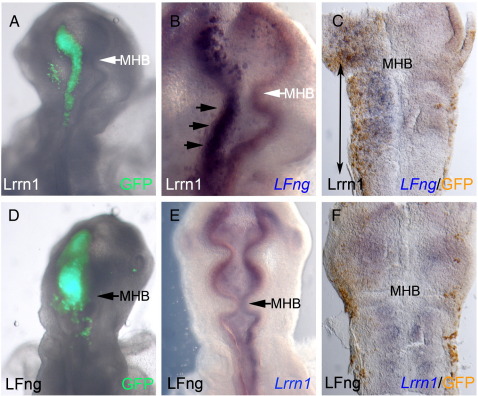
Lrrnl regulates LFng expression at the midbrain-hindbrain border (A–C) Misexpression of Lrrnl-IRESGFP or (D–F) LFng-IRESGFP. (A,D) Overlay of live fluorescence to reveal position of electroporated cells. (B,C) In-situ hybridisation of LFng (blue). (B) LFng is up-regulated on the electroporated left hand side compared to the control contra-lateral side (black arrows). (C) Flat mount preparation of neural tube reveals up-regulation of LFng within the ectopic domain of Lrnn1 detected by anti-GFP antibody (brown) (black double arrow). (E,F) In-situ hybridisation of Lrrnl (blue). No change in Lrrnl expression is seen following misexpression of LFng-IRESGFP in the neural tube. (F) Flat mount preparation reveals equal Lrrnl expression on both sides of neural tube. Anti-GFP antibody (brown) reveals position of ectopic LFng-IRESGFP-expressing cells. MHB; midbrain–hindbrain boundary.

In the *Drosophila* wing disc, *trn* and *fng* are independently activated by *Ap* to regulate two distinct processes required for boundary formation: cell affinity differences (*trn*), and signalling (*fng*), between dorsal and ventral compartments (Milan et al., 2001). We sought to investigate a possible relationship between LFng and Lrrn1 at the MHB. To test this we misexpressed Lrrn1-IRESGFP across the midbrain–hindbrain region at HH8+,9 and analysed *LFng* expression 24 h later. *LFng* was up-regulated to high levels throughout the domain where *Lrrn1* was ectopically expressed, both within its normal domains of expression and also throughout the metencephalon (r1/2) where it is normally absent (Fig. 6,A–C; n = 4/6). Both the control, contra-lateral side and embryos electroporated with a GFP control showed normal *LFng* expression with a clear absence of expression in r1/2 (n = 6/6; data not shown).

To test whether *LFng* was able to regulate *Lrrn1* expression reciprocally, we electroporated LFng-IRESGFP throughout the midbrain and anterior hindbrain and analysed *Lrrn1* expression. We did not observe any changes in *Lrrn1* expression on the side of the embryos ectopically expressing *LFng* (n = 9/9). *Lrrn1* was expressed in its normal domains of expression within midbrain and hindbrain, and remained absent from the metencephalic domain (Fig. 6D–F). Both the experimental and the control contra-lateral sides of each embryo looked the same, as did embryos electroporated with GFP control (n = 6/6; data not shown). The *LFng* border determines where Notch is activated at the midbrain–hindbrain interface (Tossell et al., submitted for publication). Therefore, through regulating *LFng* expression, Lrrn1 may determine the boundary at which Notch is activated in the neural tube, thus providing a link between compartmentalisation due to affinity differences and inter-compartmental boundary formation.

### Lrrn1 does not promote cell aggregation in transfected cells

Previous studies have provided conflicting data about the role of tartan-like LRR proteins as homophilic cell adhesion molecules. Whilst Shinza-Kameda et al. find that caps promotes homophilic cell adhesion in vitro, work from Stephen Cohen's group found no evidence of homophilic interactions but favour the hypothesis that caps and trn act by recognising an as yet unknown ligand on cells in the dorsal compartment of the wing disc (Milan et al., 2005; Shinza-Kameda et al., 2006).

To test the possibility that Lrrn1 may promote the integrity of the midbrain compartment through a homophilic cell adhesion mechanism, we assayed the ability of Lrrn1 to promote cell aggregation in HEK293T cells. A closely related LRR family member, fibronectin-leucine-rich transmembrane 3 (FLRT3), has recently been shown to promote the sorting out of transfected from non-transfected cells in this assay, a process which requires the LRR but not the intracellular domain (Karaulanov et al., 2006).

**Fig. 7:**
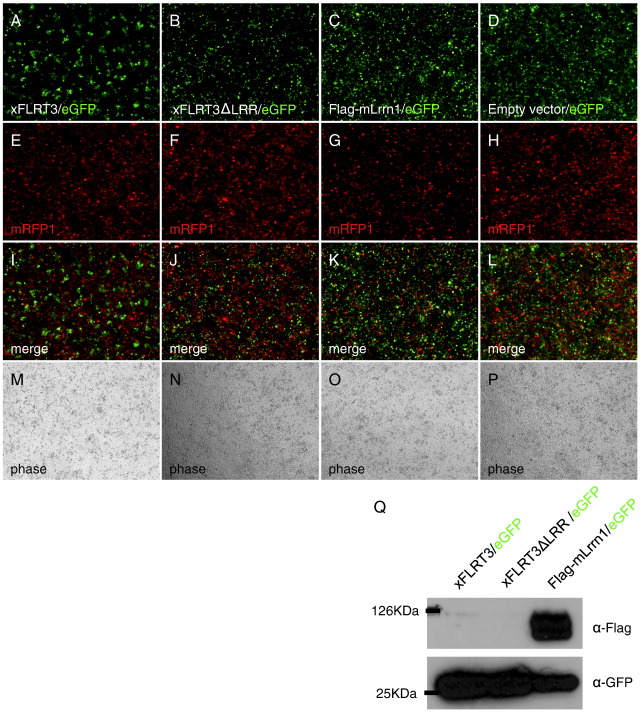
Lrrnl does not act through homophilic adhesion. HEK293T cells were cotransfected with an eGFP expression vector and constructs for (A) xFLRT3, (B) xFLRT3∆LRR (C) Flag-mLrnn1 or empty pCS2+ vector (D) aid mixed in a 1:1 ratio with control cells transfected with an mRFP1 expression vector (E–H). After 48h, xFLRT3- expressing cefis (positive control) sorted into a small, clearly defined dusters. No sorting was observed with xFLRT3∆LRR (negative control), which lacks the LRR domain of xFLRT3 necessary for homophilic interaction aid cell sorting. Sorting was not observed with Flag-tagged mLrnn1, empty vector or mRFP1 cells as can be seen in the merged nages (I–L). None of the constructs affected cell proliferation or cell attachment and confluent cell monolayers were seen in all cases under phase contrast illumination (M–P). The Flag-mLrrn1 construct was highly expressed in HEX293T cells and generated a protein of approximately 100 KDa, consistent with the size of the glycosylated form of mLrrn1 when analysed by western blot (Q).

Cells were transfected with both Lrrn1 and eGFP expression vectors at a ratio that ensured effective co-transfection of the two plasmids (9:1). These were then mixed with mRFP1 transfected cells and allowed to form aggregates (Karaulanov et al., 2006). In contrast to FLRT3/eGFP transfected cells, which sorted in to small, clearly defined clusters over a 48 h period (Fig. 7A,E,I), Lrrn1/GFP transfected cells formed a mixed population with RFP transfected cells and did not exhibit any detectable sorting activity (Fig. 7C,G,K). Identical results were obtained with Lrrn1-IRESGFP, Lrrn1T-GFP, Lrrn2 and Lrrn3 (data not shown). Therefore, Lrrn1 does not appear to promote homophilic cell adhesion in the same way as FLRT3, suggesting that the affinity differences between Lrrn1 expressing midbrain cells and Lrrn1 non-expressing hindbrain cells is modulated indirectly, by an as yet unidentified mechanism.

## Discussion

*Lrrn1* is the vertebrate orthologue of *Drosophila trn*, a gene involved in DV boundary formation in the wing disc through specification of dorsal cell affinity properties (Milan et al., 2001, 2005). Because the expression border of *Lrrn1* at the MHB correlates with that of *Otx2*, which defines the midbrain compartment, we investigated the hypothesis that Lrrn1 may play a similar role to trn in boundary formation at the MHB in the vertebrate CNS. In common with the DV boundary, Notch signalling is important for the formation of the MHB, where it specifies boundary cell fate (Tossell et al., submitted for publication). Furthermore, the co-expression of *Lrrn1* and *LFng* in the midbrain compartment is strikingly similar to that seen with *trn/fng* in the dorsal compartment of the *Drosophila* wing disc.

### The role of Lrrn1 in cell restriction and compartition at the MHB

Both loss and gain of function experiments resulted in a disruption of the MHB. Blocking Lrrn1 function at the MHB resulted in a loss of the morphological constriction at the MHB and in some cases, complete loss of the organiser gene, *Fgf8*; probably as a secondary consequence of loss of the boundary. Ectopic expression of *Lrrn1* across the MHB region resulted in a shift of the boundary (identified by midbrain markers *Otx2* and *Wnt1,* and hindbrain markers *Gbx2* and *Fgf8*), with some mixing between midbrain and hindbrain cells. Cell labelling confirmed that cell mixing was due to a lack of restriction of midbrain cells, which were able to cross into anterior hindbrain within the ectopic *Lrrn1* domain. The shift in position of the boundary is probably due to the cell mixing observed. This suggests that Lrrn1 acts to restrict cells at the boundary, by mediating an affinity difference between midbrain and hindbrain cells that prevents cells from mixing at their interface. Similarly, overexpression of *trn* at the tarsus5/pretarsus boundary of the *Drosophila* leg disc causes cells to violate compartmental restriction and cross into the adjacent domain, suggesting that Lrrn1 may perform a similar function to trn at vertebrate boundaries (Sakurai et al., 2007).

### Integration of segregation, boundary formation and organiser signalling at the MHB

At the DV boundary in the *Drosophila* wing disc, affinity differences between D and V cells are initially regulated by caps/trn independently of signalling at the boundary, which is mediated by Notch. The selector gene *Ap* directs both cell affinity and cell signalling at the DV boundary through independent regulation of both *caps/trn* and *fng* (Milan and Cohen, 2003; Milan et al., 2001). We have found that *Lrrn1* upregulates *LFng* in the hindbrain and, therefore, it may determine the border of *LFng* expression at the posterior midbrain where they are co-expressed. In a similar manner to the DV boundary in the wing disc, the *LFng* border (in concert with the expression border of the Notch ligand *Ser1*) plays an important role at the MHB to determine where Notch is activated (Tossell et al., submitted for publication). Unlike the situation in the wing disc, however, the regulation of *LFng* by Lrrn1 may provide a mechanism for linking compartmentalisation, due to affinity differences, and signalling, at inter-compartmental boundaries.

It is possible that a vertebrate orthologue of *Ap* also functions to regulate both *LFng* and *Lrrn1* at the MHB, providing a mechanism for linking compartmentalisation, due to affinity differences, and signalling, at inter-compartmental boundaries. Indeed, one family member, *Lmx1b*, is expressed broadly in the forebrain and midbrain at HH9, becoming restricted to midbrain at HH10 with a posterior boundary of expression that coincides with *Otx2* and *Wnt1* (Yuan and Schoenwolf, 1999). In the chick, *Lmx1b* acts upstream of *Wnt1* and *Fgf8* to induce and maintain MHB organiser genes (Adams et al., 2000; Matsunaga et al., 2002). Furthermore, mouse and zebrafish mutant analyses have revealed that *Lmx1b* is essential for the proper formation of the MHB organiser, and later tectum and cerebellum development (Guo et al., 2007; O'Hara et al., 2005).

We have found a close correlation between the onset of *Fgf8* expression and the down-regulation of *Lrrn1* at the MHB, and indeed have shown that FGF8 protein represses *Lrrn1* expression in midbrain. Thus, a complex interaction is becoming apparent that ensures the precise positioning of the boundary between midbrain and hindbrain. Initially, *Otx2* and *Gbx2* are expressed broadly across anterior and posterior neural plate, respectively (Millet et al., 1996; Wassarman et al., 1997). From 3 somites (HH8−), MHB genes are expressed in an initially broad domain covering posterior midbrain and anterior hindbrain. *Fgf8* itself is expressed from 4 somites (HH8) (Shamim and Mason, 1999). Correlating with this, a down-regulation of *Lrrn1* is observed shortly after, from 7 somites (HH9) in the *Fgf8* domain, at a time when organiser genes are broadly expressed prior to boundary formation and before restriction to the boundary. Given that recombinant FGF8 protein represses *Lrrn1* it is likely that the normal down-regulation of *Lrrn1* in anterior hindbrain (r1) is due to repression by FGF8. The domain over which *Lrrn1* is repressed by an FGF8 bead is striking. Previously, inductive responses to FGF8 have not been observed to cross rhombomere boundaries, yet strikingly, here we observed *Lrrn1* repression across a number of rhombomere boundaries (Irving and Mason, 2000).

### How does Lrrn1 function at the MHB?

We propose that Lrrn1 provides an affinity cue for cells in the midbrain, which sharpens and defines the boundary by segregating *Lrrn1*-positive cells from the cells that have turned off *Lrrn1* expression in r1. At the same time, Lrrn1 integrates boundary cell determination by the Notch signalling pathway, through the regulation of *LFng*. This leads to a sharp *LFng* border which acts to define the domain of Notch activation, and subsequently where specialised boundary cells are positioned (Tossell et al., submitted for publication).

Given the results of our cell aggregation assay it is unlikely that the segregation of midbrain and hindbrain cells occurs through a homophilic adhesion mechanism, although it is possible that Lrrn1 binds homophilically, but requires the addition of a co-receptor which is missing from the HEK293 cell line. Previously, aggregation assays have been described for other members of the LRR family, such as caps (Shinza-Kameda et al., 2006) and FLRT3 (Karaulanov et al., 2006), which showed that these family members can bind homophilically. However, although FLRT3 indeed demonstrates the capacity to bind homophilically in our assay, Lrrn1 does not.

FLRT3 functions by regulating the levels of cadherins on the surface of cells via endocytosis, and has also been identified as a co-receptor for FGF (Bottcher et al., 2004; Haines et al., 2006; Karaulanov et al., 2006). *FLRT3* is also expressed at the MHB and it is possible that it regulates both cell adhesion and FGF signalling there. However, co-transfection of Lrrn1 and FLRT3 was unable to block the homophilic aggregation of FLRT3-expressing cells in our assay (data not shown), suggesting that it does act independently of FLRT3, perhaps in a parallel pathway.

We have also been unsuccessful in our attempts to identify a receptor, or ligand, for Lrrn1, using a number of strategies (Andreae et al., 2007). Therefore it is likely that Lrrn1 forms part of a multiprotein complex with other co-receptors, or binds to either a complex or small molecule ligand. Indeed, the crystal structure of another LRR protein, Lingo-1, reveals that it forms a tetramer which acts as a scaffold to assemble the Nogo receptor complex (Mosyak et al., 2006).

## Figures and Tables

**Table 1:** FGF8 represses *Lrrn1* expression.

	FGF8 bead	PBS bead
*Lrrn1*	5/7	0/9
*Fgf8*	2/2	0/2
